# Electronic and ionic effects of sulphur and other acidic adsorbates on the surface of an SOFC cathode material†

**DOI:** 10.1039/d3ta00978e

**Published:** 2023-03-15

**Authors:** Matthäus Siebenhofer, Andreas Nenning, George E. Wilson, John A. Kilner, Christoph Rameshan, Markus Kubicek, Jürgen Fleig, Peter Blaha

**Affiliations:** a Centre for Electrochemistry and Surface Technology, CEST Wr. Neustadt Austria matthaeus.siebenhofer@tuwien.ac.at; b Institute of Chemical Technologies and Analytics, TU Wien Vienna Austria; c Department of Materials, Imperial College London UK; d Chair of Physical Chemistry, Montanuniversität Leoben Leoben Austria; e Institute of Materials Chemistry, TU Wien Vienna Austria

## Abstract

The effects of sulphur adsorbates and other typical solid oxide fuel cell (SOFC) poisons on the electronic and ionic properties of an SrO-terminated (La,Sr)CoO_3_ (LSC) surface and on its oxygen exchange kinetics have been investigated experimentally with near ambient pressure X-ray photoelectron spectroscopy (NAP-XPS), low energy ion scattering (LEIS) and impedance spectroscopy as well as computationally with density functional theory (DFT). The experiment shows that trace amounts of sulphur in measurement atmospheres form SO^2−^_4_ adsorbates and strongly deactivate a pristine LSC surface. They induce a work function increase, indicating a changing surface potential and a surface dipole. DFT calculations reveal that the main participants in these charge transfer processes are not sub-surface transition metals, but surface oxygen atoms. The study further shows that sulphate adsorbates strongly affect oxygen vacancy formation energies in the LSC (sub-)surface, thus affecting defect concentrations and oxygen transport properties. To generalize these results, the investigation was extended to other acidic oxides which are technologically relevant as SOFC cathode poisons, such as CO_2_ and CrO_3_. The results unveil a clear correlation of work function changes and redistributed charge with the Smith acidity of the adsorbed oxide and clarify fundamental mechanistic details of atomic surface modifications. The impact of acidic adsorbates on various aspects of the oxygen exchange reaction rate is discussed in detail.

## Abstract 2

A combined experimental and computational approach reveals electronic and ionic effects of acidic adsorbates on mixed conducting oxide surfaces.

## Graphical abstract

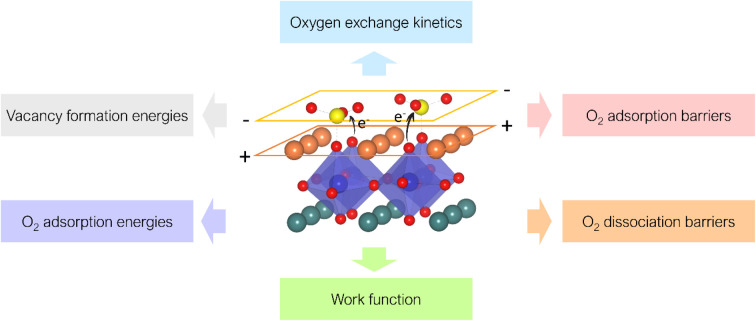

## Introduction

Solid oxide fuel and electrolysis cells (SOFCs/SOECs) are promising representatives of innovative and clean electrochemical energy-conversion technologies. They pose interesting opportunities for a variety of applications due to their high efficiency and their ability to operate on a range of different hydrocarbon fuels.^1–4^ While cell performance steadily increases and new materials are being developed to drive SOFCs towards lower operating temperatures,^5–9^ a key hindrance for a wide applicability lies in their inherent susceptibility to performance degradation, in particular with regard to the oxygen exchange reaction (OER) on the cathode side.^10–12^

Apart from morphological degradation processes such as delamination or cracking, cathode degradation factors can be broadly divided into two categories: (1) materials change chemically under operating conditions, affecting the catalytic activity of the surface (*e.g.* Sr segregation in Sr-containing perovskites);^13–15^ (2) environmental impurities (*e.g.* S, CO_2_ or Cr) accumulate on the cathode and poison its surface, leading to second phase formation and to a performance decrease.^16–19^ Regarding the atomic mechanism of degradation processes, many suggestions have been brought forward, ranging from the blocking of active reaction sites by adsorbates^20^ or secondary phases^17,21^ to element depletion or accumulation in (sub-)surface regions of the material.^22^

In a recent study, the authors could show that trace amounts of sulphur, omnipresent in measurement gases in the high ppb range^23^ and also in air in the low ppb range,^18,24,25^ readily adsorb on the surface of La_0.6_Sr_0.4_CoO_3−*δ*_ (LSC), a highly active but degradation prone SOFC cathode material.^23^ There, they form SO^2−^_4_ surface species and cause a sudden, strong drop of the OER rate. The study also revealed that this phenomenon is not unique to Sr-containing perovskites but also occurs on other potential SOFC cathode materials, such as Pr_0.1_Ce_0.9_O_2−*δ*_. Further investigations identified this SO^2−^_4_ formation as the starting point of long-term degradation processes in the form of second phase formation which are commonly associated with Sr segregation.^26^ In conjunction with recent advances identifying the acidity of oxidic surface modifications as indicative for their catalytic activity,^27^ these results strongly emphasize the importance of the outermost surface for the final performance of a material. However, while it is possible to qualitatively predict the surface activity of modified surfaces based on their acidity, the atomistic mechanism behind such modifications is still unclear.

The present study investigates in detail the effects of acidic adsorbates on the LSC surface and discusses their implications for the OER kinetics. Utilizing near-ambient pressure X-ray photoelectron spectroscopy (NAP-XPS) and low energy ion scattering (LEIS), a detailed picture of the surface chemistry is drawn. Based on these experimental findings, *ab initio* calculations are employed to examine charge redistribution processes at the surface of LSC upon acidic adsorbate formation. Supported by density functional theory (DFT), the impact of acidic adsorbates on the electronic and ionic properties of LSC, as well as on the adsorption of molecular oxygen is discussed, illustrating various effects of surface modifications on the OER kinetics.

## Methodology

### Experimental methods

#### Sample preparation

LSC thin films were prepared *via* pulsed laser deposition (PLD) on (001) oriented yttria-stabilized zirconia (YSZ) single crystals (9.5% Y_2_O_3_, 5 × 5 × 0.5 mm, Crystec GmbH, Germany). For an oriented growth of the LSC thin films, a Gd_0.2_Ce_0.8_O_2−*δ*_ (GDC) buffer layer was prepared by PLD (X-ray diffraction data is presented in S.3 of the ESI†).^28,29^ PLD parameters are given in Table 1, the film thickness (50 nm) was calibrated with a quartz balance in the PLD chamber. For electrochemical measurements during NAP-XPS, a Ti/Pt grid (5 nm Ti, 100 nm Pt, 25/5 μm holes per grid) was prepared on both sides of the single crystals by photolithography and ion beam etching. Depending on the analysis method, different preparation routes for the back sides of the samples were chosen. For NAP-XPS measurements, a fast, nanoporous LSC counter electrode was deposited *via* PLD (parameters are given in Table 1).^30^ On samples for LEIS measurements, the single crystal backsides were painted with Pt paste to ensure constant heat absorption during PLD. For all PLD depositions, a KrF excimer laser with a fluence of 1.1 J cm^−2^ was used (Compex Pro 201F, 248 nm, Coherent), the substrates were heated on a resistive heating stage and the temperature was checked at the beginning of the deposition with an infrared pyrometer (Heitronics, Germany).

**Table 1:** PLD parameters for the deposition of dense and porous LSC thin films

Material	LSC dense	LSC porous	GDC
Substrate-target distance (cm)	6.0	5.0	6.0
Background O_2_ pressure (mbar)	0.04	0.4	0.04
Temperature (°C)	600	450	600
Frequency (Hz)	2	5	1

#### Near ambient pressure X-ray photoelectron spectroscopy

NAP-XPS measurements were performed in a lab-based setup using a PHOIBOS NAP photoelectron analyzer (SPECS, Germany) with a monochromated Al K-α XR 50 MF (microfocus) X-ray source. During experiments, samples were mounted on a custom sample holder with a 4.5 × 4.5 mm^2^ hole directly below the sample for laser heating with a near-infrared diode laser.^31^ The samples were held in place by Pt–Ir needles which also served as electrical contacts. A pyrometer was used to measure the sample temperature during the measurements. At 400 °C, precise temperature control is possible also by evaluation of the high-frequency ohmic resistance of the impedance spectrum, which corresponds to the well known temperature dependent YSZ conductivity^32^ and which was used to calibrate the sample emissivity. Spectra were recorded in 8 × 10^−6^ mbar O_2_ (5.0 purity, Messer GmbH, Austria) at 600 °C. For temperature control, impedance spectra were recorded with an Alpha-A High Performance Frequency Analyzer and Electrochemical Test Station POT/GAL 30 V/2 A setup (Novocontrol Technologies, Germany). To achieve a measurable sulphate contamination, the pressure was increased to 1 mbar. This procedure leads to a non-negligible concentration of sulphur compounds in the measurement atmosphere, sufficient to warrant sulphate adsorption on LSC surfaces.^23^ XPS spectra were recorded at an analyzer pass energy of 30 eV. For investigations of the work function, the low energy cut-off of inelastically scattered electrons was determined. A sample bias of −20 V was applied in order to slightly accelerate the very low energy electrons towards the analyzer. For better energy resolution and adequate count rate, an analyser pass energy of 5 eV and an energy step width of 0.02 eV were used. Due to the low electron energy and long pathway in the NAP analyser, precisely tuned active magnetic shielding was mandatory.

#### Low energy ion scattering

To probe the outermost atomic layer of the surface, low energy ion scattering (LEIS) measurements were performed using a QTAC 100 LEIS device (IONTOF GmbH, Germany). Atomic signals were measured with a 3 keV ^4^He^+^ primary analysis beam at a 90° incidence angle. The analyzed area was 1 × 1 mm^2^ and a beam current of 5 nA was used. Measurement statistics were improved by 5 collection rounds for each measurement. Prior to measurement, two cleaning methods were investigated separately; first, samples were cleaned by reactive oxygen plasma cleaning in a preparation chamber for 20 min, secondly, the samples were heated to 400 °C in the analysis chamber (residual pressure ≈10^−9^ mbar) at a rate of 2 °C min^−1^. Depth profiles were obtained by sputtering at a 60° incidence angle with a 500 eV ^40^Ar^+^ beam and a beam current of 100 nA.

### Computational methods

**Fig. 1:**
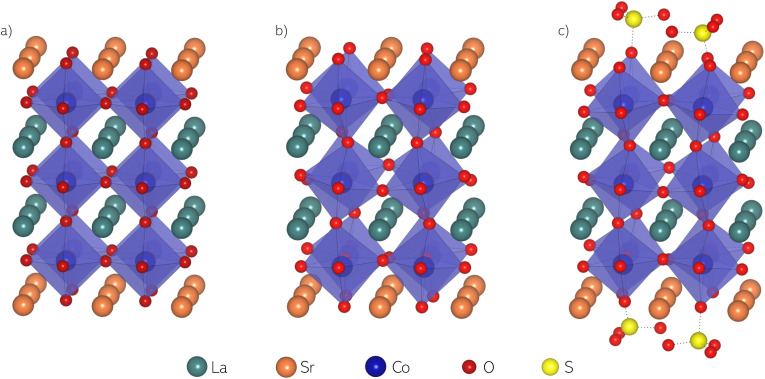
(a) Unrelaxed slab model of LSC with a full SrO termination layer; (b) relaxed slab model of LSC with a full SrO termination layer (LSC-S0); (c) relaxed slab model of LSC with a full SrO termination layer and two sulphate adsorbates on each surface (“half-coverage”, LSC-S2).

DFT calculations of (La,Sr)CoO_3−*δ*_ were performed using the full-potential augmented plane wave plus local orbitals method^33^ as implemented in the WIEN2k code.^34,35^ For all calculations, the PBE GGA (generalized gradient approximation) functional^36^ was used. For a suitable treatment of the correlated 3d-electrons of Co and Cr atoms, a Hubbard U correction was employed, using a potential *U*_eff_ = *U* − *J* of 3.35 eV for both atoms, considering previous investigations of similar materials.^37–39^ A volume optimization for LaCoO_3_ yielded a pseudocubic lattice parameter of 3.83 Å, being in good agreement with experiment.^40,41^ As a base model for all calculations we used a symmetric 2 × 2 × 3 LaO terminated (001) surface slab of LaCoO_3_ with the top La atoms replaced by Sr (see Fig. 1). Thereby, not only the stoichiometry is corrected towards realistic LSC cathodes with the La : Sr : Co ratio being 1 : 1 : 1.5 (1 : 0.7 :  1.7 for La_0.6_Sr_0.4_CoO_3−*δ*_), the model also considers the experimentally found SrO termination of LSC under solid oxide cell operating conditions.^42,43^ A symmetric slab was chosen over a two termination structure to avoid overall dipole formation which might interfere with electrostatic effects induced by adsorbates. Overall, the slab contains 8 Sr, 8 La, 12 Co and 40 O atoms. A vacuum region of 20 Å separates the slabs to minimize interactions between the surfaces.^44,45^

During calculations, different surface modifications were placed on the LSC slab, an exemplary structure with two sulphate adsorbates is shown in Fig. 1 c. To find the most stable configurations, a structure relaxation was performed for all structures, employing a *Γ*-centered 5 × 5 × 1 k-mesh. For a valid comparison of total energies, final calculations were performed with the same basis-set size corresponding to *R*^min^_MT_*K*_max_ = 6, with *R*^min^_MT_ being the smallest atomic sphere radius (2.2, 2.2, 1.87, 1.15, 1.15, 1.38 and 1.38 bohr for La, Sr, Co, O, C, S and Cr atomic spheres respectively) and *K*_max_ the largest reciprocal lattice vector. All relaxations were performed until residual forces did not exceed 3 mRy per bohr for three consecutive iterations. As energy separation between core and valence states, −6.0 Ry was used.

Depending on the environmental conditions, *i.e.* temperature and p(O_2_), the charge imbalance introduced by Sr-doping in LSC can be compensated electronically or by oxygen vacancies (V_O_). To investigate the interplay of acidic adsorbates and oxygen vacancies in the LSC surface and the LSC bulk, single oxygen atoms were removed from the structure and structure relaxation was performed. For a closer investigation of oxygen adsorption, O_2_ molecules were placed in and above the surface. Energetics of adsorption and oxygen vacancy formation have been evaluated by comparing the total energies of the final structures with the sum of the constituent structures, *e.g.* for O_2_ adsorption: 
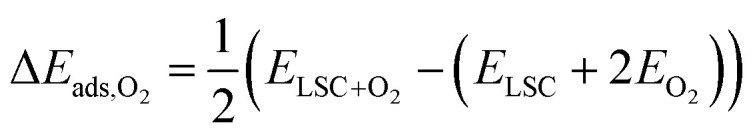
. The factors of 1/2 and 2 arises from the symmetric slab with two surfaces. DFT calculations of O_2_ and SO_2_ molecules have been performed with the same *R*^min^_MT_*K*_max_ and *R*_MT_ values to ensure compatibility of the total energy values.

To quantitatively assess charge transfer upon adsorption of acidic molecules or O_2_, Bader's quantum theory of atoms in molecules was used.^46^ Here, a charge is assigned to every atom in the slab, whereas the atom is defined as the region bounded by a zero flux surface 
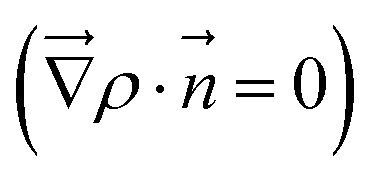
, and the electron density *ρ* is integrated over this region. To differentiate between geometric and electronic effects, the results are compared with a superposition of neutral atoms.

For investigations of the adsorption barrier for O_2_, an O_2_ molecule was placed far above a surface V_O_ (O–Co distance ∼4–6 Å). From this starting point, a constraint minimization technique was employed, slowly reducing the distance between the lower O and the Co below by applying a pseudoforce on both atoms. Thereby, adsorption processes can be simulated and adsorption barriers can be estimated.

## Results and discussion

### Investigation of real LSC surfaces

#### X-ray photoelectron spectroscopy

**Fig. 2:**
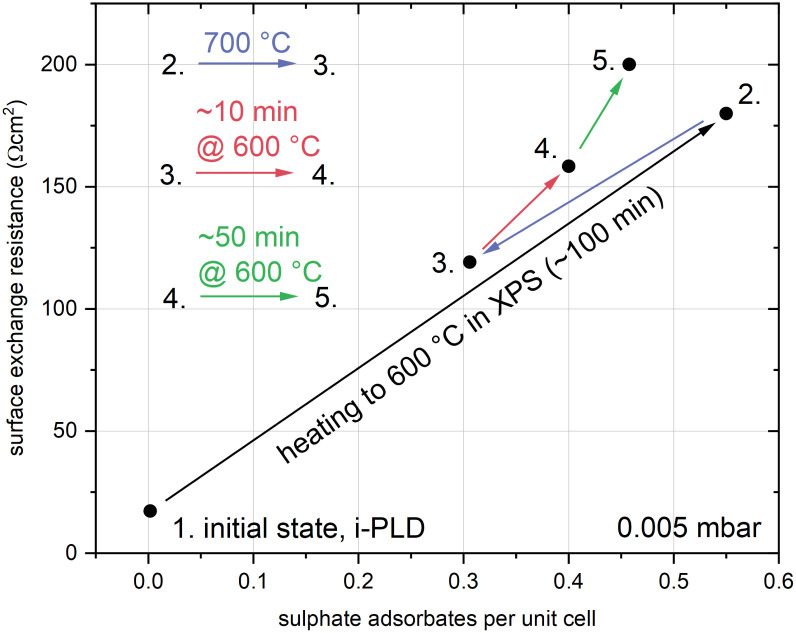
Evolution of the surface coverage with sulphate adsorbates and the corresponding change of the surface exchange resistance on a dense, (100) oriented LSC thin film with respect to temperature and time in 0.005 mbar O_2_. Sulphate groups were quantified from XPS measurements.^23^

Building on recent findings where omnipresent trace amounts of sulphur impurities in the measurement gas were identified as the reason for a sudden and substantial performance degradation of LSC from its pristine state,^23^ further XPS investigations were performed. A summary of the absolute effect of sulphate adsorbates on the surface exchange resistance is shown in Fig. 2. Starting with no S signature on a sample directly out of the PLD (the surface exchange resistance was evaluated with i-PLD^23^), heating to 600 °C in 0.005 mbar O_2_ leads to a significant formation of sulphate species on the surface, accompanied by a strong increase of the resistance by nearly a factor of 10. Heating to 700 °C decreased the amount of S, in accordance with an adsorbate model, and, after cooling back to 600 °C, also the resistance had decreased. Further measurements at 600 °C showed again an increase of S in combination with a resistive increase.

**Fig. 3:**
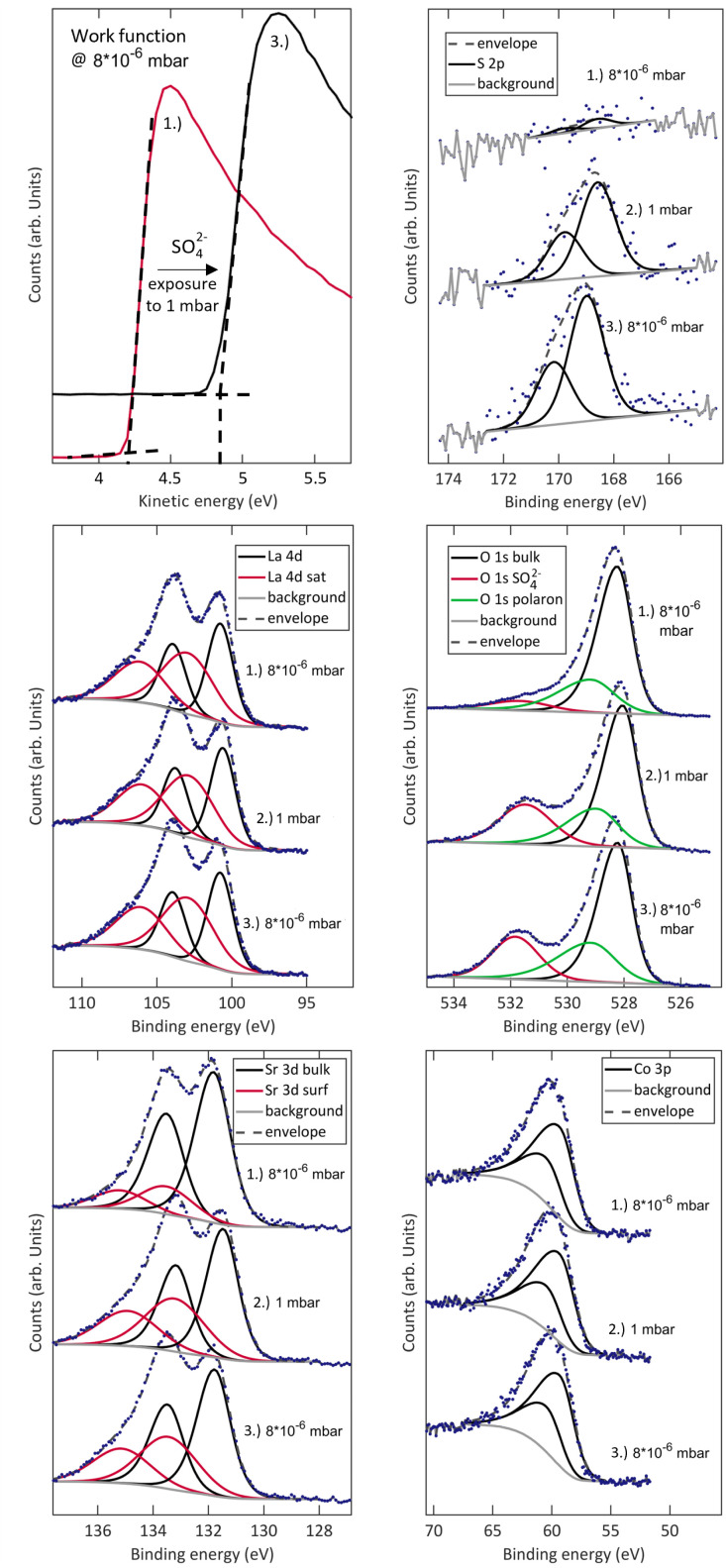
NAP-XPS measurements of S 2p, La 4d, O 1s, Sr 3d, Co 3p and the work function of a (001)-oriented dense LSC thin film at 8 × 10^−6^ mbar O_2_ and 600 °C before (1.) and after (3.) exposure to 1 mbar of O_2_ at 600 °C (2.).

The detailed effects of sulphate adsorbates on the surface chemistry of LSC are shown in Fig. 3. XPS measurements were first performed at 600 °C and 8 × 10^−6^ mbar O_2_, warranting very low impurity contents. Then, the samples were exposed to 1 mbar of O_2_, where an immediate saturation with sulphate adsorbates is obtained. The signals of La, Sr and Co remain nearly unchanged, indicating that the effect of sulphate adsorbates is not directly related to significant changes of Co oxidation states or the cation stoichiometry on the surface. The signals of O and S evolve as expected for sulphate formation with the sulphur signal increasing together with the appearance of an oxygen satellite feature at 532 eV, which has already been attributed to S-related compounds in the past.^23,47^ A ratio of S 2p and 532 eV O 1s atomic fractions of 0.23 agrees excellently with SO^2−^_4_ species.^23^ The slight shift of the S 2p species observed upon changing the oxygen partial pressure is likely correlated with a different surface potential induced by different oxygen vacancy concentrations and oxygen adsorbate coverages at different p(O_2_).

While this evolution of the surface chemistry has already been reported, a closer investigation of low kinetic energy regions reveals an additional change induced by SO^2−^_4_ adsorbate formation. The same measurement routine of measuring a clean sample at 8 × 10^−6^ mbar O_2_, allowing sulphates to accumulate at 1 mbar and measuring again at 8 × 10^−6^ mbar O_2_ unveils a significant change of the work function from 4.2 to 4.8 eV. This is a clear indication for the formation of a charged adsorbate layer and a space charge in the material. As a very rough estimate for the reach of such electrostatic effects, the Debye length can be calculated by^48^1
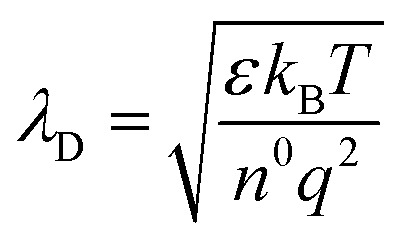
with *ε* denoting the permittivity of the material (for LSC a value of 20 is assumed^49^), *k*_B_ the Boltzmann constant, *T* the temperature, *n*^0^ the dopant concentration and *q* the dopant charge. Due to the high doping level of La_0.6_Sr_0.4_CoO_3−*δ*_, *λ*_D_ amounts only to ≈ 0.1 nm, indicating that the reach of electrostatic effects is very limited. However, elastic effects might surpass this reach and translate into sub-surface regions. At this point it is noteworthy that this work function change also occurs on other structurally and chemically different oxides, such as Pr_0.1_Ce_0.9_O_2−*δ*_ (see S.2 in the ESI†). While this study is focused on LSC as a cathode material, we strongly suspect that the fundamental processes unveiled here also hold for other cathode materials.

#### Low energy ion scattering

For a detailed investigation of the outermost surface layer, low energy ion scattering (LEIS) measurements were performed. With this method, only the first atomic layer of the surface is probed.^50,51^ Two different samples were investigated by LEIS: one pristine LSC sample directly out of the PLD chamber and one LSC sample, which was exposed to 1 mbar of O_2_ (5.0 purity, Messer Austria) at 600 °C in the NAP-XPS chamber, where the surface is saturated with sulphate adsorbates (identical to XPS measurements).

**Fig. 4:**
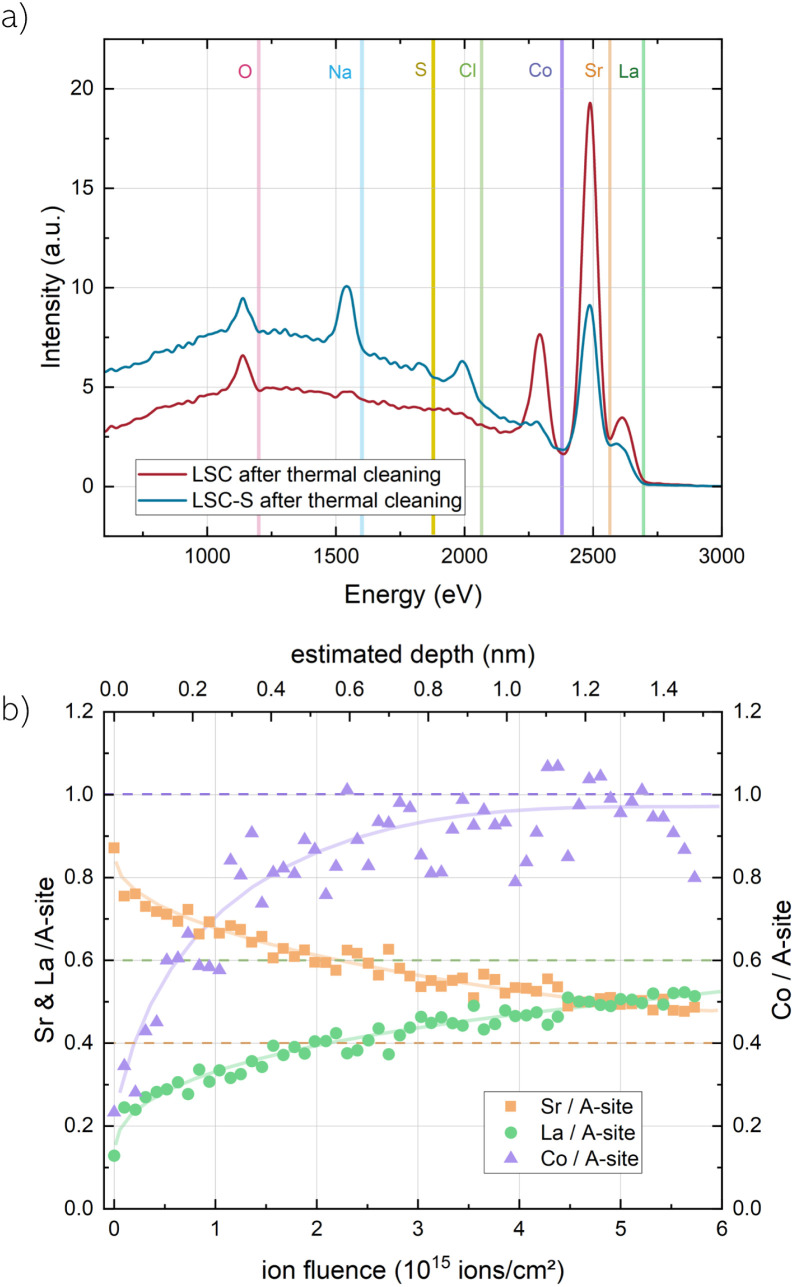
(a) LEIS spectra (3 keV ^4^He^+^ ions, 5 nA) of LSC directly out of the PLD chamber and sulphate-covered LSC-S (after exposure to 1 mbar O_2_ in the XPS chamber at 600 °C) after thermal cleaning by *in situ* heating to 400 °C. Vertical lines show the onset scattering energy of each surface atom. (b) depth-profiles of La, Sr and Co for the first 1.5 nm of LSC-S obtained by sputtering with a 500 eV ^40^Ar^+^ beam with a beam current of 100 nA. Signals were normalized to the bulk stoichiometry of a reference measurement in greater depth.

Prior to measurement, the samples were exposed to high vacuum (≈10^−9^ mbar) overnight, for an initial surface cleaning by desorption of loosely bound surface adsorbates. In addition, the samples were exposed to an *in situ* heating step to 400 °C (2 K per minute) to remove adventitious carbonates from the surface. The resulting LEIS spectra are shown in Fig. 4 and are denoted as LSC(-S) after thermal cleaning. For both samples, Sr clearly dominates the surface cation distribution, even more so for sulphate covered LSC. The measurements also yield the first direct proof of sulphur on the surface, albeit with a rather weak intensity. We strongly suspect that this is due to the SO^2−^_4_ environment which shields the sulphur from approaching ions. Additionally, NaCl traces appeared on the surface (much more pronounced on LSC-S2, where they are likely also the cause for the generally lower main cation intensities). We suspect that these traces stem either from contaminations on the heating stage or from handling during sample transfer. Na is also a common impurity found in oxide materials and very small amounts suffice to yield detectable contamination in LEIS measurements.^52^

In Fig. 4b, a depth profile of the cation ratios in the first 1.5 nm of a sulphate covered LSC thin film is shown (cation ratios were quantified by identifying the plateau reached after sufficient sputtering with the ideal bulk stoichiometry^51^). Similar to earlier investigations of LSC with LEIS,^43^ the results emphasize the Sr enrichment and show a strong Co depletion in the first layer, as well as a broader La depletion zone. It is worth mentioning that preferential sputtering will alter the ion yield,^53^ chiefly affecting lighter species, such as oxygen. Therefore, the measurements will slightly overestimate the La content in the LSC surface, however, this effect is difficult to correct for and we only suspect negligible deviations due to the low ion fluence. While these measurements illustrate that real LSC surfaces can be rather complicated, the results agree with NAP-XPS measurements and also with computational findings which are described below:

– LSC exhibits a Sr-rich termination layer and the surface is depleted of Co an La.

– LSC-S is covered with acidic sulphate adsorbates, which are bound in an SO^2−^_4_ environment and are surrounded by oxygen atoms, likely accounting for the low sulphur signal.

Summarizing the surface analytical results of this study, NAP-XPS and LEIS investigations agree with the model that real LSC surfaces are largely SrO terminated and that sulphur readily adsorbs in an SO^2−^_4_ environment, likely associated with surface Sr. We find that SO^2−^_4_ formation leads to a significant shift of the LSC work function, indicating the formation of a surface dipole. In the following, we will employ DFT calculations to obtain a detailed understanding of the impact of SO^2−^_4_ adsorbates on the electronic and ionic structure of LSC.

### *Ab initio* calculations of SO^2−^_4_ adsorbates on LSC

To understand the detailed effects of sulphate adsorbates on the electronic and ionic properties of LSC, *ab initio* calculations have been performed with WIEN2k.^34,35^ We investigated stable structures with different coverages of sulphate adsorbates and examined energetic and electrostatic effects. In particular, we evaluated adsorption energies of SO_3_ and O_2_ molecules on SrO-terminated LSC surfaces, the adsorption barrier for an approaching O_2_ molecule, as well as oxygen vacancy formation energies. Electrostatic effects were investigated by a Bader charge analysis,^46^ by evaluating work function changes upon SO_3_ adsorption and by analyzing the densities of states of specific atoms. The results of these calculations not only clarify the experimentally found surface dipole formation but also illustrate the manifold ways acidic adsorbates affect the oxygen exchange reaction.

#### Energetics of adsorbates and vacancies

##### SO^2−^_4_ adsorbate structures and adsorption energies

The foundation for all calculations was a relaxed LSC surface slab with a full SrO termination (LSC-S0, see Fig. 1b). The structure exhibits a clear octahedral tilting, in agreement with earlier experimental studies on LSC.^54^ Moreover, a slight surface buckling is visible with the oxygen atoms positioned ≈0.2 Å above the Sr atoms. Starting from there, SO_3_ groups were placed directly above a surface oxygen and structure relaxation calculations were performed. The relaxed structure is shown in Fig. 1c. S–O bond distances and O–S–O bond angles in the sulphate adsorbate range between 1.46–1.48 Å and 112–117° for the three top oxygens and between 1.62–1.64 Å and 103–104° for the bottom oxygen in the LSC surface, respectively. Upon sulphate adsorption, the bottom oxygen gets slightly pulled out of the surface by ≈0.2 Å and the buckling increases correspondingly. As a consequence, the Co–O bond length to the subsurface also increases by the same 0.2 Å at sulphate covered sites. The octahedral tilting of the LSC structure gets stronger upon sulphate adsorption with the tilting angle increasing from 6° to 10° (final coordinates for different structures are given in S.1†). The core-level shift to higher binding energies observed in the O 1s species of bulk LSC and SO^2−^_4_ during XPS is reproduced by the calculations but slightly underestimated (2.5 eV *vs.* 3.5 eV experimentally). Calculated adsorption energies of SO_3_ groups for LSC with a 25% (LSC-S1) and a 50% sulphate coverage (LSC-S2), as well as for LSC-S2 with a surface vacancy are given in Table 2. As adsorption mechanism we suggest the adsorption of SO_2_ and the subsequent oxidation to SO_3_. Therefore, the reaction enthalpy for the oxidation reaction 
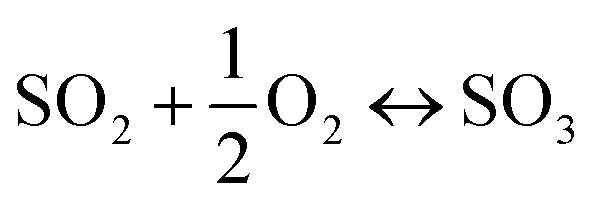
 of −1.025 eV^55^ was subtracted from the energy difference: 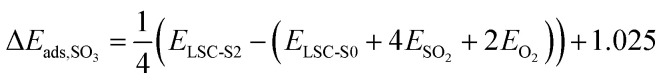. The results show that SO_3_ groups are very strongly adsorbed on SrO-terminated LSC, with the adsorption strength decreasing with increasing coverage. For reduced surfaces with oxygen vacancies, an even stronger adsorption is found.

**Table 2:** Adsorption energies of one SO_3_ anion compared for LSC-S1, LSC-S2 and LSC-S2 with a surface vacancy

	LSC-S1	LSC-S2	LSC-S2 + V_O_
Adsorption energy per SO_3_ (eV)	−3.31	−2.46	−2.96

The high calculated adsorption energies are also in line with experimental results which show that it is very hard to remove sulphate adsorbates from LSC surfaces.^23^ A decrease of sulphate coverage can be observed at 0.005 mbar O_2_ at a temperature of 700 °C, but sulphate groups adsorbed at 600 °C tend to remain on the surface. With regard to mitigation strategies, it is particularly interesting that samples directly after the PLD process do not exhibit sulphur signatures during XPS measurements. Instead, after transfer in ambient air, the surfaces are covered with substantial amounts of adventitious carbonates and we strongly suspect that these act as a protective layer against sulphates. This might indicate the possibility to modify SOFC cathode materials towards a sulphur resistant surface, potentially utilizing compounds which are only slightly acidic relative to the host lattice.

##### Vacancy formation energies

To assess the effects of SO^2−^_4_ adsorbates on the ionic properties of LSC surfaces, vacancy formation energies have been investigated. Oxygen vacancies (V_O_) have been created in the outermost surface (V_O,surf_, placed on a free surface O site for LSC-S2) and in the LaO layer (V_O,bulk_) and the total energies of the relaxed structures were compared. The resulting formation energies are given in Table 3.

**Table 3:** Oxygen vacancy formation energies per V_O_ in the surface and the “bulk” (LaO layer) of LSC-S0 and LSC-S2

	Surface (eV)	“Bulk” (eV)
LSC-S0	1.30	1.12
LSC-S2	0.19	2.15

Regarding the importance of V_O_ for the oxygen exchange kinetics,^56–58^ this result requires further discussion. From an electrostatic point of view, it is reasonable that a negatively charged SO^2−^_4_ layer promotes the formation of positive V_O,surf_, as charge compensation is limited to short distances, evident by the small formation energy for surface vacancies on LSC-S2. However, the correlation of vacancy concentration and oxygen exchange kinetics has been topic of an ongoing discussion.^59,60^ Moreover, the much larger formation energy for a bulk vacancy might indicate a certain blocking of vacancy migration between the bulk and the surface of LSC. The absolute impact of surface modifications on the reaction rate will be discussed in a broader context below, including electrostatic as well as chemical contributions.

##### O_2_ adsorption energies

O_2_ adsorption energies have been evaluated for an O_2_ molecule placed in a V_O,surf_. The results are shown in Table 4, revealing a substantial difference between LSC-S0 and LSC-S2. While for the sulphur free LSC-S0, the adsorption of an O_2_ molecule is energetically favourable, it costs energy to place an oxygen molecule between the sulphate groups while annihilating a surface vacancy. In addition, the bond distance of the O_2_ molecule is considerably increased on LSC-S0 (1.43 Å *vs.* 1.34 Å on LSC-S2), possibly indicating inhibited dissociation of the molecule on LSC-S2.

**Table 4:** Adsorption energies for one O_2_ molecule on LSC-S0 + V_O_ and LSC-S2 + V_O_

	LSC-S0 + V_O_	LSC-S2 + V_O_
Adsorption energy per O_2_ (eV)	−1.40	0.48

##### O_2_ adsorption barriers

**Fig. 5:**
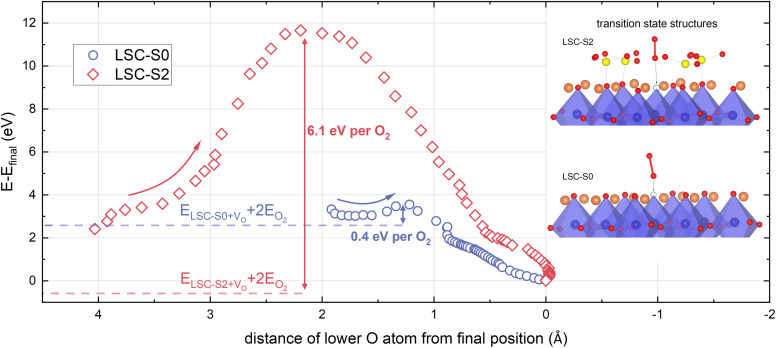
Evolution of the total energy during the approach of an O_2_ molecule to its final position in a surface vacancy on LSC-S0 and LSC-S2. The energy barriers are indicated in the graph and transition state structures are shown in the inset. The curves consist of ≈1300 (LSC-S0) and ≈1800 (LSC-S2) data points and the data has been averaged over 30 points. For barrier calculations, an *R*^min^_MT_*K*_max_ value of 5 was used for higher throughput, accounting for minor differences in adsorption energies.

Next to electrostatic and energetic changes, sulphate adsorbates might also affect the adsorption barrier of an O_2_ molecule. While this study does not aim for a realistic modelling of O_2_ adsorption processes on LSC surfaces, DFT calculations allow for a qualitative assessment of changing adsorption barriers upon sulphate adsorption. For this purpose, an O_2_ molecule was placed far away from the surface and pulled towards its final position (see computational methods). The total energy was recorded as a function of the distance to the final position of the O_2_ molecule (see Fig. 5).

The method itself makes use of different simplifications. The O_2_ molecule does not possess initial momentum, all atoms are in static equilibrium positions and a pseudo-force is exerted on the lower O atom and the Co atom below (which might actually mimic oscillating of the atoms in a real adsorption situation) which is fully omitted after the barrier maximum is passed. Still, the analysis of the energy barrier illustrates that sulphate adsorbates change the energy landscape during O_2_ adsorption drastically. The energy barrier for this specific adsorption process changes from 0.4 eV to 6.1 eV per adsorbed O_2_ molecule. Also, the adsorption barrier occurs at a much larger distance from the LSC-S2 surface compared to LSC-S0.

##### O_2_ dissociation pathways

The dissociation of an O_2_ molecule in a surface vacancy on LSC-S0 and the subsequent lateral migration of the dissociated O atom were investigated computationally. Three different dissociation pathways were examined: dissociation *via* (i) a Sr bridge position between two nearest neighbour surface O atoms, (ii) a Sr bridge position in the second direction (these two directions differ by their orientation towards the tilted O-octahedra), (iii) a Sr top position, between two next nearest O atoms (relaxed structures are shown in S.2†). The dissociated O atom was constrained in *x* and *y* direction and structure relaxation was performed. The energy differences between the molecular O_2_ adsorbate and the dissociated structure give an indication about energetically favourable dissociation paths (Table 5).

**Table 5:** Energy barriers for the dissociation of an adsorbed O_2_ molecule on LSC

	Sr bridge 1	Sr bridge 2	Sr top
Δ*E* (eV)	1.60	1.68	2.95

According to the calculations, the least likely dissociation path proceeds on top of a surface Sr atom. Instead, the dissociated atom is more likely to move between two surface Sr atoms. For the calculated structures of LSC-S2, these favourable dissociation pathways between two Sr atoms are blocked by SO^2−^_4_ adsorbates and thus only the energetically unfavorable dissociation path *via* the Sr top position remains, however, we also expect a higher dissociation/migration barrier due to the steric hindrance caused by SO^2−^_4_ adsorbates.

#### Surface dipole formation and charge redistribution

##### Work functions

As the work function is the prime experimental indication for the formation of a surface potential and a surface dipole, it has also been evaluated computationally for sulphate covered SrO-terminated LSC surfaces. The work function was calculated as the difference between the Fermi energy of the surface slab and the vacuum coulomb potential for the electron, evaluated in the middle of the vacuum between two neighbouring slabs. The results are shown in Table 6 and are in qualitative agreement with the experimental results. Starting with a relatively low work function, it increases steadily upon sulphate adsorption. Quantitatively, the experiment shows an increase of the work function from 4.2 to 4.8 eV for a SO^2−^_4_ coverage of ≈50%. However, a direct comparison of experimental and computational results is not straightforward, as XPS measurements were performed at elevated temperature at 8 × 10^−6^ mbar O_2_. Instead of a highly idealized surface with a perfectly (001) oriented SrO termination with no steps or kinks, we expect a surface with certain reconstructions, a substantial amount of surface vacancies, especially on sulphate covered LSC (which decrease the work function by supplying electrons, see also Table 6), and a non-negligible surface roughness. To exclude a dependency of the work function on the vacuum size, the work function of LSC-S0 was recalculated with a bigger vacuum (+20 bohr), yielding a value of 3.65 eV, demonstrating the validity of the analysis. In conclusion, theory confirms the experimental results that sulphate adsorption leads to a substantially increased work function. It is worth mentioning, that a similar phenomenon has already been discussed in the context of surface acidity and an extension to other acidic oxides will be discussed below .^27^

**Table 6:** Work functions of pristine and sulphate covered LSC surfaces

	LSC-S0	LSC-S1	LSC-S2	LSC-S2 + V_O_
WF (eV)	3.69	6.18	7.65	6.78

##### Charge redistribution

**Fig. 6:**
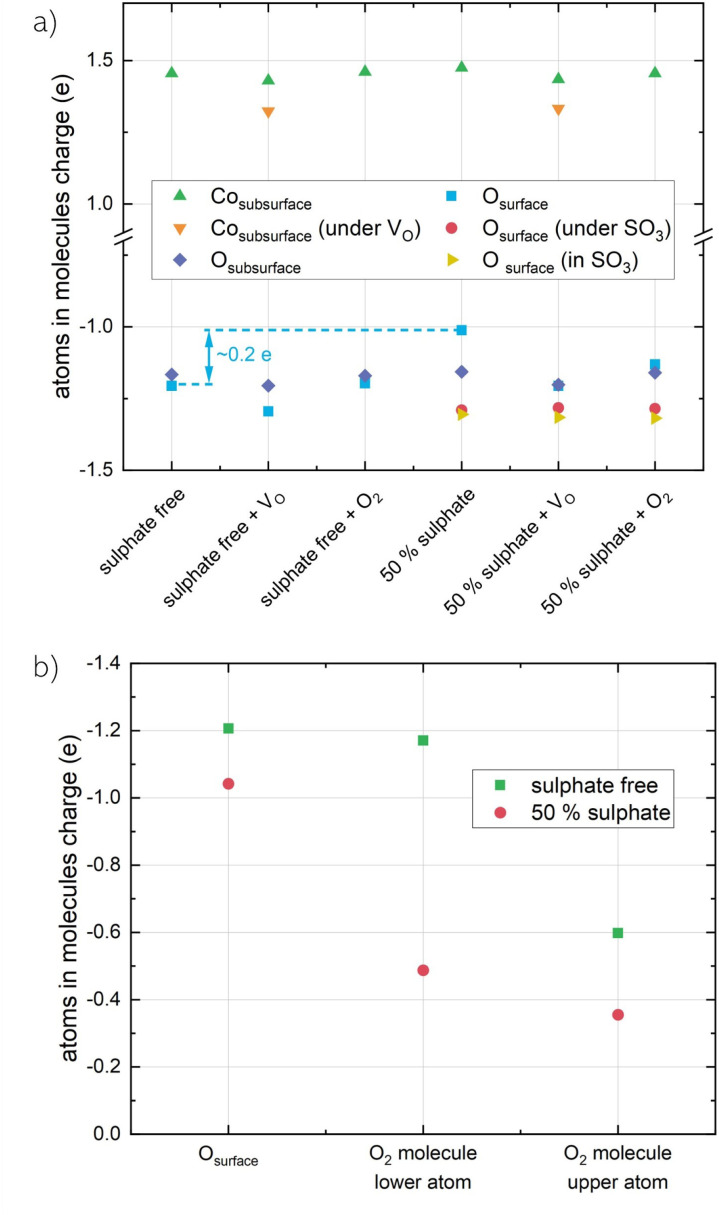
(a) Bader charges of subsurface Co atoms and surface O atoms for different scenarios. (b) Bader charges of O atoms in an adsorbed O_2_ molecule for LSC-S0 and LSC-S2 surfaces.

As was shown experimentally and confirmed computationally, surface adsorption of sulphur results in the formation of SO^2−^_4_ surface species and an increase of the work function, indicating the formation of a surface dipole and charge transfer at the surface. To further investigate such charge transfer processes, the atomic charges in LSC-S0 and LSC-S2 have been compared using Baders theory of atoms in molecules.^46^ Selected results of this analysis are shown in Fig. 6a. Surprisingly, the charge of subsurface Co atoms is hardly affected by sulphate formation on the SrO-terminated LSC surface. Instead, a significant change of the charge of surface oxygens can be observed upon the addition of SO_3_ groups. According to the analysis, free surface oxygens transfer substantial amounts of charge towards oxygen in the sulphate group, leading to a charge depletion in the free outermost surface and a negative charge accumulation in the sulphate adsorbates. For LSC-S2, a charge of ∼0.2 *e* per free surface oxygen is transferred from the surface towards the adsorbates. With regard to the range of these redistribution effects, LSC-S1 was investigated as well, where only one of the 4 surface oxygens in the cell is covered with an SO_3_ group. Here, only the 2 nearest oxygen neighbours donate charge towards the adsorbate, while the diagonal oxygen is not affected. It is also noteworthy that, during all Bader charge investigations, the La charges remained completely unaffected.

In addition, a Bader charge analysis was also performed for structures containing a surface vacancy and structures with an O_2_ molecule adsorbed in the surface vacancy. For vacancy containing structures, the additional two electrons introduced by the surface vacancy are distributed across both, O and Co atoms, indicating that polaronic charge carriers in LSC are not exclusively localized on Co and that O atoms play a substantial role in charge compensation in LSC. This is also in line with a recent tendency to assign electron holes in La-based perovskites a strong oxygen character and to accredit surface oxygen a high importance during charge transfer steps in catalytic redox reactions.^61–67^

This is further confirmed by the Bader charge analysis for an adsorbed O_2_ molecule on LSC-S0 and LSC-S2 (see Fig. 6b). The amount of charge which is transferred to the O_2_ molecule is substantially higher for the sulphate free surface than it is for the sulphate covered surface, underlining the importance of surface O for charge transfer during the oxygen reduction reaction. This result is also in line with previous investigations, describing the formation of superoxo species in oxygen vacancies on AO-terminated surfaces of different mixed conducting perovskites (ABO_3_) and Ruddlesden-Popper materials (A_2_BO_4_).^68,69^ Interestingly, the difference between the charge localized at the adsorbed oxygen molecule on LSC-S0 compared to LSC-S2 amounts to 0.93 e, possibly establishing a tie to mechanistic discussions of the oxygen exchange reaction where single electron transfer is often considered as an essential reaction step.^57,70^

**Fig. 7:**
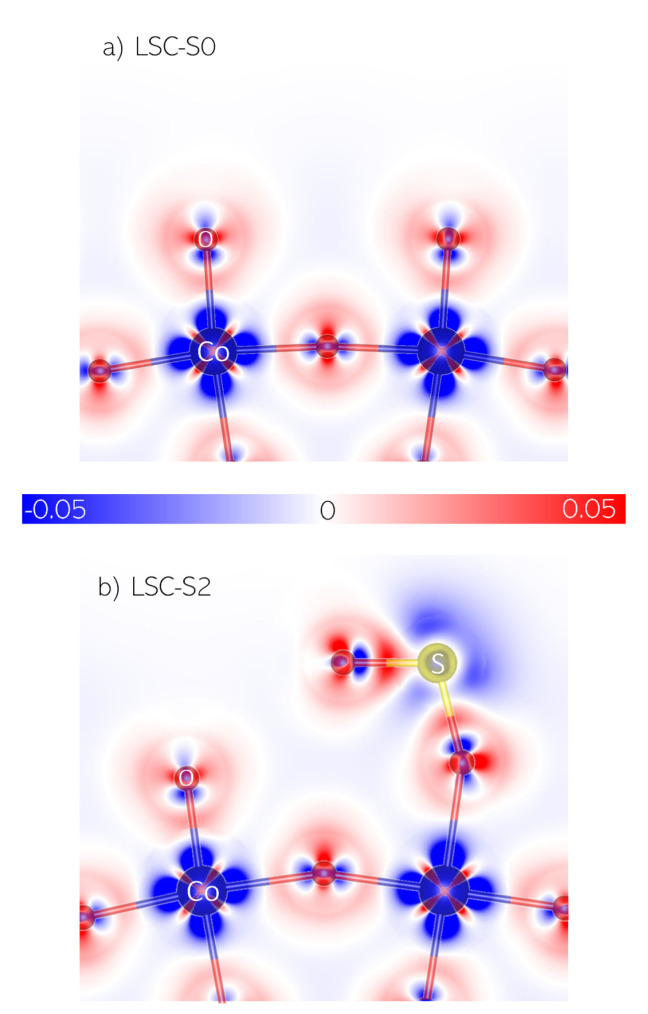
Difference electron density maps of a vertical CoO_2_ plane for LSC-S0 (a) and LSC-S2 (b). Blue regions denote less electron density relative to the neutral lattice.

The spatial distribution of charge is also visualized in difference density plots in Fig. 7, *i.e.* the difference of the self consistent electron density and the superposition of spherical neutral atoms densities. Here, apart from the orbital configuration and the strong covalent contribution to the Co–O bonds, one can observe a stronger buckling of the sulphate-covered surface as well as the strong bond between the SO_3_ group and the surface atom below, forming the SO^2−^_4_ adsorbate.

##### Densities of state (DOS)

**Fig. 8:**
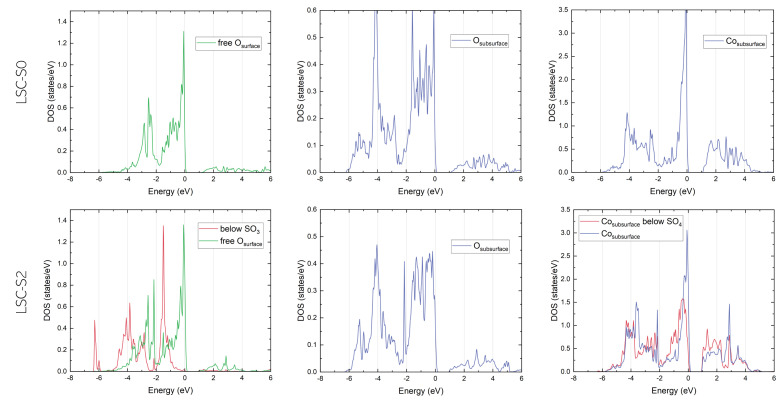
Densities of state for O-2p and Co-3d electrons for sulphate-free (top) and sulphate-covered (bottom) surfaces.

For more information about the electrostatic effects induced by SO^2−^_4_ adsorbates, the partial density of states (PDOS) of different surface and subsurface atoms on LSC-S0 and LSC-S2 was investigated. In particular, the PDOS of O 2p and Co 3d electrons were examined (Fig. 8), as these are the prime candidates to be affected by sulphate adsorption. A first examination of the results confirms the picture suggested by the Bader charge analysis – while the Co DOS is only slightly affected when SO_3_ is adsorbed on the LSC surface, the surface O PDOS change substantially. In particular, a strong shift to lower energies for the O 2p state can be observed (it is also noteworthy that the O 2p bandwidth at the surface with 4 eV is significantly narrower than in the subsurface with 6 eV).

This shift of the O 2p band is especially interesting in light of the ongoing discussion about the suitability of the O 2p band center as a descriptor for the oxygen reduction activity of a mixed conducting surface.^71–74^ For the present calculation, adsorption of an SO_3_ group moves the O 2p band center (identified as the centroid position of the O 2p band) from −1.44 eV on the sulphate free surface to −1.59 eV for a free surface O atom on a sulphate-covered surface and to −2.75 eV for an O below an SO_3_ group. Considering that reduction of molecular oxygen requires charge transfer to oxygen in a surface vacancy, it seems reasonable that this shift of the O 2p band of potentially charge transferring surface oxygens indeed contributes to a decrease of the reaction kinetics.

At the same time, these results also call for a critical examination of the idea of such a descriptor for the oxygen exchange activity. Due to the strong effect on the oxygen exchange kinetics observed upon sulphate adsorption (and surface modification in general^27^), it is questionable if the bulk O 2p band center is able to describe the oxygen activity of a material beyond a truly pristine state. Additionally, several studies have demonstrated the importance of surface terminations for the total oxygen exchange activity which is also not reflected by a bulk descriptor.^44,51,75^ Therefore, we suggest to further investigate the upper band edge of O atoms of a specific surface structure, which might more accurately describe the energetics of the oxygen reduction reaction.

Summarizing the so far obtained results, it becomes clear that sulphate adsorbates affect the surface properties of SrO-terminated LSC in manifold ways. The main underlying process upon sulphate adsorption is a charge transfer from surface oxygen towards the SO^2−^_4_ adsorbates. This introduces a surface dipole which is reflected in a substantial increase of the LSC workfunction, as was also observed experimentally. Additionally, sulphate adsorbates strongly affect the adsorption of O_2_ molecules, both *via* the adsorption barrier and *via* the final configuration of the adsorbate itself, affecting charge transfer and potentially also dissociation steps.

### Effects of different acidic adsorbates

Apart from sulphur, several other oxides are known to degrade the oxygen exchange kinetics on SOFC cathode materials, such as CO_2_^20,76,77^ or CrO_3_.^78,79^ A common property of all these oxides is their acidity relative to usual SOFC cathode material surfaces.^27,80–82^ While this fact has already been recognized previously,^27,81^ no conclusive mechanistic explanation for the correlation of surface acidity and oxygen exchange kinetics has been brought forward yet. While a detailed experimental and theoretical investigation of different combinations of surface oxides and host materials is far beyond the scope of this study, we suspect that the mechanism behind the here investigated sulphate induced degradation plays a major role in this discussion.

**Fig. 9:**
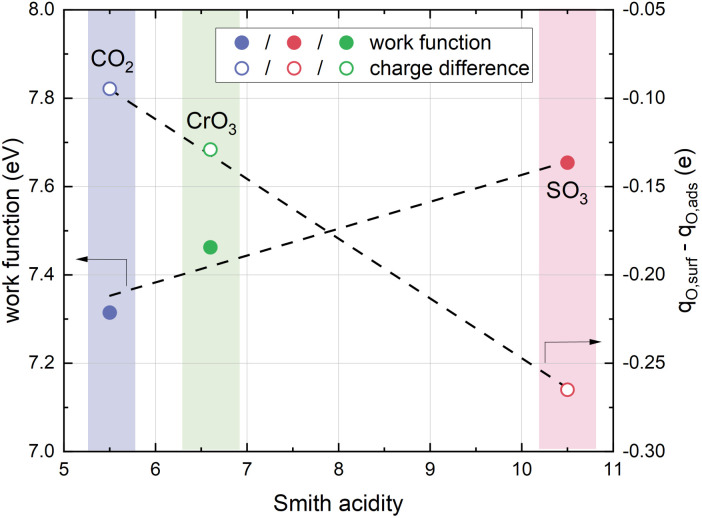
Effect of different acidic adsorbates on the work function and on the charge difference between surface oxygen atoms and adsorbate oxygen atoms on SrO-terminated LSC surfaces.

For an initial assessment, the investigation is extended towards two other different, technologically relevant, acidic oxides, which may adsorb on the SrO-terminated LSC surface instead of SO_3_ groups. Computationally, SO_3_ groups have been replaced by CO_2_ and CrO_3_ groups, leading to carbonate (CO^2−^_3_) and chromate (CrO^2−^_4_) adsorbates. After a structure relaxation, the charge transfer has been assessed by performing a Bader charge analysis and calculating the difference between the charge of a free surface O atom and an adsorbate O atom. Again, Co atoms in the subsurface are not affected, confirming earlier investigations on SO_3_ groups. In addition, the work function has been evaluated for the CO_2_ and CrO_3_ covered LSC. The results are shown in Fig. 9. Very interestingly, we find that both the transferred charge and the work function correspond very well to the Smith acidity^80^ of the respective oxides, strongly indicating that the suggested phenomenon of charge transfer and surface dipole formation truly is the underlying process upon adsorption of acidic oxides.

In addition, the formation energies of oxygen vacancies on acidic surfaces have been evaluated, with the results shown in Table 7. Again, computational results confirm that the qualitative behaviour of sulphate covered LSC translates to other acidic adsorbates, *i.e.* the formation energy of surface oxygen vacancies is reduced substantially compared to the unperturbed SrO-termination. Quantitatively, the correlation with the Smith acidity is not as clear as for charge transfer and work function, in particular CrO^2−^_4_ adsorbates yield a low vacancy formation energy compared with SO^2−^_4_ adsorbates. The reason for this, however, requires further investigation of the LSC-CrO_3_ system.

**Table 7:** Oxygen vacancy formation energies per vacancy in the surface of LSC-S0 and SrO-terminated LSC covered with sulphate, carbonate and chromate adsorbates

	LSC-S0	CO^2−^_3_	CrO^2−^_4_	SO^2−^_4_
V_O,surf_ formation energy (eV)	1.30	0.47	0.18	0.19

### Impact of acidic adsorbates on the oxygen exchange reaction kinetics

To correlate theoretical predictions with experimental findings, it is necessary to discuss the impact of acidic adsorbates on the oxygen exchange kinetics in more detail. While significant advances have been achieved recently with regard to the clarification of the oxygen exchange mechanism,^44,56,57,70,83^ there is still no final consensus about the specific roles of the participating defects in the single reaction steps. It appears agreed upon that oxygen vacancies play an essential role in the incorporation of molecular oxygen, however, the discussion about whether charge transfer occurs before, during and/or after dissociation is still ongoing. Therefore, the here presented discussion tentatively assumes that charge transfer is coupled to the binding environments of the adsorbed oxygen molecule/atom and that the oxygen incorporation reaction follows the scheme (i) molecular adsorption (ii) dissociation (iii) incorporation.

Based on the experimental and computational results we suggest that the following aspects need to be considered when discussing the oxygen exchange kinetics on LSC surfaces covered with acidic adsorbates:

– Acidic adsorbates affect the adsorption energy of O_2_ molecules as well as the adsorption barrier, thus strongly affecting upstream reaction steps of the incorporation direction. The same might hold for upstream reaction steps of the oxygen evolution direction, as the concentration of surface vacancies combined with a high acidic adsorbate coverage might strongly affect the number of surface oxygens available for O_2_ molecule formation. A more in-depth discussion of the connection between equilibrium concentrations and reaction energetics is presented in S.3.†

– If the dissociation of adsorbed O_2_ is the rate limiting step of the incorporation direction (different O_2_ dissociation pathways are discussed above), the kinetic barrier of this step will be affected by acidic adsorbates, indicated by the smaller bond length and reduced charge of the adsorbed O_2_ on LSC-S2 as well as different migration barriers or blocked dissociation pathways.

– Defect concentrations in and beneath the LSC surface are altered considerably. Oxygen vacancies clearly accumulate on surfaces with acidic adsorbates, however, subsurface vacancies are very unfavourable. This might strongly affect the oxygen transport to and from the surface. The situation for electronic charge carriers is even more complicated, as it depends on which particular species participates in charge transfer to the O_2_ molecule. While the oxygen exchange reaction is commonly written under the assumption that charge transfer equals a reduction or oxidation of a metal cation (*e.g.* Co), as mentioned earlier, recent studies indicate that electron holes have a strong oxygen character.^62,67^ While DFT calculations suggest that transition metals are not affected substantially by acidic adsorbates, for the latter case, with oxygen participating in the charge transfer, acidic adsorbates will have a strong effect on the availability of charge carriers for the reaction.

– Lastly, depending on the mechanism of the oxygen exchange reaction, also the surface dipole/potential itself, which is established by the charge transfer to the adsorbates will have an impact on the final reaction rate if charge is transferred across this electric field.

## Conclusions

The electronic and ionic effects of acidic adsorbates on (La,Sr)CoO_3−*δ*_ (LSC) surfaces have been investigated experimentally and computationally. Near ambient pressure XPS and low energy ion scattering have been employed to examine the surface chemistry of LSC surfaces upon exposure to sulphur containing trace impurities in the measurement atmosphere. The experiment found that sulphur is bound in an SO^2−^_4_ environment and that these adsorbates induce a significant shift of the work function by 0.6 eV. Impedance spectroscopy revealed that such sulphate formation processes lead to a strong and sudden degradation of the oxygen surface exchange kinetics in equilibrium. *Ab initio* calculations were then performed to gain insight into the detailed processes occurring on LSC surfaces upon sulphate adsorption. These calculations found strongly bound sulphate adsorbates which cause various changes in the surface chemistry of LSC: (i) charge is transferred from surface oxygen atoms to the sulphate groups; (ii) vacancy formation energies are significantly reduced in a sulphate covered surface and increased in the sub-surface; (iii) sulphate adsorbates inhibit the adsorption of molecular O_2_ into a surface vacancy by strongly increasing the adsorption barrier and by rendering an O_2_ adsorbate configuration energetically unfavourable. Exemplary calculations have been performed for two additional acidic adsorbates CO^2−^_3_ and CrO^2−^_4_ and reveal a quantitative agreement of electrostatic changes (amount of transferred charge and work function changes) with the Smith acidity scale, which has recently been proposed as a descriptor for the oxygen exchange kinetics on mixed conducting surfaces. Several processes have been identified which might affect the oxygen exchange kinetics of LSC surfaces in contact with acidic adsorbates, expanding our understanding of mixed conducting surfaces and the oxygen exchange reaction itself.

## Conflicts of interest

There are no conflicts of interest to declare.

## Supplementary Material

TA-011-D3TA00978E-s001
