# Supplementary material for: Electronic and ionic effects of sulphur and other acidic adsorbates on the surface of an SOFC cathode material
Source: J Mater Chem A Mater. 2023 Mar 15;11(13):7213–26. doi: 10.1039/d3ta00978e (PMC10044886; doi:10.1039/d3ta00978e)
Supplement: TA-011-D3TA00978E-s001 [file TA-011-D3TA00978E-s001.pdf]

Electronic Supplementary Material (ESI) for Journal of Materials Chemistry A.
This journal is © The Royal Society of Chemistry 2023

Electronic and ionic effects of sulphur and other acidic adsorbates on the surface of an SOFC cathode material

Matthäus Siebenhofer, Andreas Nenning, George E. Wilson, John A. Kilner, Christoph Rameshan, Markus Kubicek, Jürgen Fleig, Peter Blaha

S.I. 1. equilibrium structures

In the following section, the essential equilibrium structures used in the main manuscript are shown with the corresponding fractional coordinates. For every atom, a second counterpart atom exists on the inverse position (e.g. for 0.1/0.1/0.1, a second atom with the coordinates 0.9/0.9/0.9 exists). The supercell dimensions for most calculations were 7.66 x 7.66 x 32.62 Å.

LSC with SrO termination and no adsorbates:

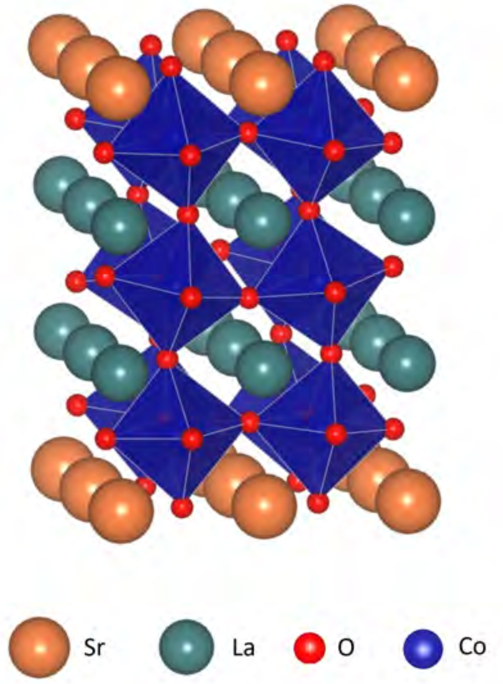

			x	y	z
1	Sr	Sr1	0.99918	0.99834	0.32962
3	Sr	Sr3	0.49932	0.0024	0.32923
5	Sr	Sr5	0.99918	0.50205	0.32959
7	Sr	Sr7	0.49932	0.49805	0.32928
9	La	La1	0.00028	0.9972	0.55933
11	La	La3	0.5002	0.00175	0.55972
13	La	La5	0.00022	0.50308	0.55935
15	La	La7	0.5003	0.4986	0.55969
17	Co	Co1	0.74964	0.75	0.37996
19	Co	Co3	0.24905	0.75002	0.37988
21	Co	Co5	0.25066	0.74999	0.62024
23	Co	Co7	0.75037	0.75001	0.62015
25	Co	Co9	0.74929	0.75001	0.4999
27	Co	Co11	0.25075	0.75	0.49989
29	O	O1	0.99939	0.74938	0.38974
31	O	O3	0.24873	0.00006	0.38555
33	O	O5	0.00051	0.7491	0.62064
35	O	O7	0.74999	0.00009	0.38561
37	O	O9	0.50049	0.75066	0.61119
39	O	O11	0.25149	0.50007	0.6145
41	O	O13	0.49935	0.75088	0.37737
43	O	O15	0.74983	0.50004	0.61439
45	O	O17	0.75095	1	0.49989
47	O	O19	0.00004	0.74976	0.48943
49	O	O21	0.75033	0.5	0.50018
51	O	O23	0.50004	0.75028	0.51078
53	O	O25	0.76514	0.75017	0.32236
55	O	O27	0.23275	0.74996	0.32232
57	O	O29	0.26365	0.75009	0.67779
59	O	O31	0.73739	0.74988	0.6777
61	O	O33	0.78568	0.75033	0.55794
63	O	O35	0.21449	0.74967	0.55794
65	O	O37	0.28897	0.75028	0.44181
67	O	O39	0.71107	0.74963	0.44182

LSC with SrO termination and one SO_4^{2-} adsorbate:

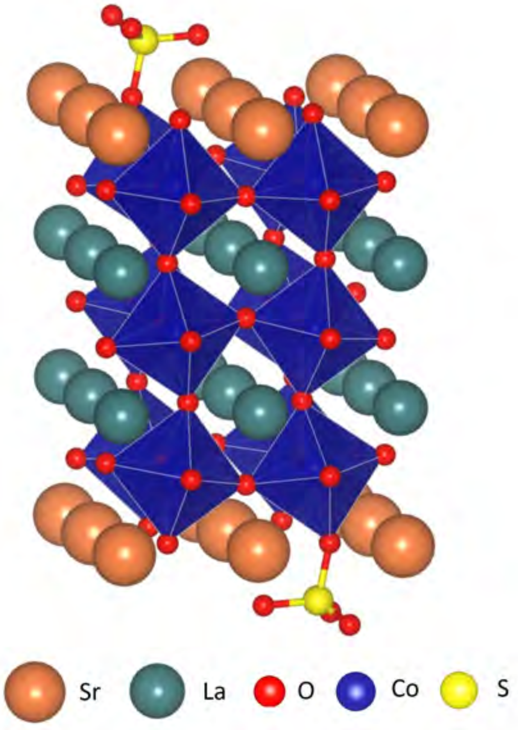

			x	y	z
1	Sr	Sr1	0.99175	0.98657	0.67403
3	Sr	Sr3	0.50927	0.99311	0.67463
5	Sr	Sr5	0.99177	0.51331	0.67402
7	Sr	Sr7	0.50923	0.50682	0.67461
9	La	La1	0.99959	0.00464	0.44031
11	La	La3	0.49816	0.00246	0.44007
13	La	La5	0.99963	0.49531	0.44032
15	La	La7	0.4981	0.49745	0.44008
17	Co	Co1	0.25254	0.25001	0.61671
19	Co	Co3	0.75162	0.25	0.61935
21	Co	Co5	0.74808	0.24998	0.38007
23	Co	Co7	0.25044	0.24999	0.38019
25	Co	Co9	0.24916	0.24999	0.50045
27	Co	Co11	0.74837	0.25	0.50042
29	O	O1	0.001	0.25012	0.60693
31	O	O3	0.74867	0.99982	0.61423
33	O	O5	0.99955	0.25012	0.38046
35	O	O7	0.25031	0.99966	0.61482
37	O	O9	0.49967	0.24989	0.3889
39	O	O11	0.74939	0.49964	0.38509
41	O	O13	0.49891	0.24986	0.62827
43	O	O15	0.25155	0.49981	0.38581
45	O	O17	0.24957	0.00019	0.5007
47	O	O19	0.99977	0.25004	0.51083
49	O	O21	0.24969	0.49981	0.50054
51	O	O23	0.49913	0.24994	0.48995
53	O	O25	0.19851	0.24986	0.67856
55	O	O27	0.78024	0.25005	0.675
57	O	O29	0.73541	0.24986	0.3241
59	O	O31	0.2592	0.25005	0.32225
61	O	O33	0.21619	0.24983	0.44289
63	O	O35	0.7816	0.2502	0.44252
65	O	O37	0.70731	0.24986	0.55852
67	O	O39	0.29789	0.2502	0.55984
69	S	S1	0.24048	0.24998	0.72605
71	O	O41	0.16166	0.08655	0.74162
73	O	O43	0.16179	0.41357	0.74157
75	O	O45	0.43585	0.24995	0.72902

LSC with SrO termination and two SO_4^{2-} adsorbates:

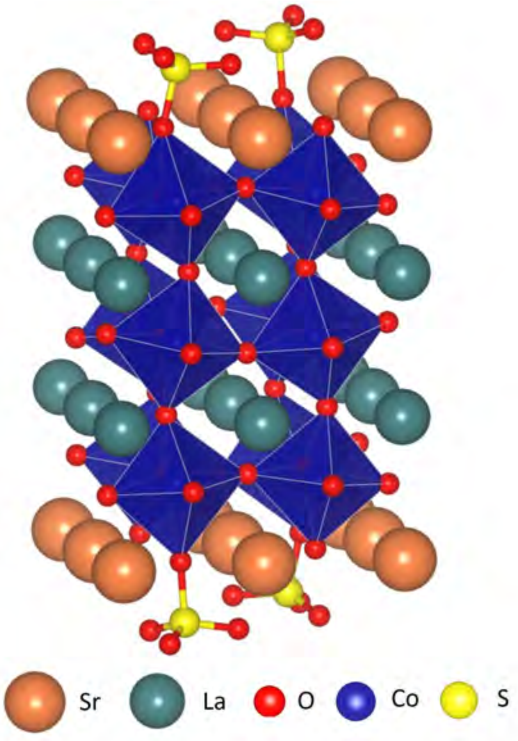

			x	y	z
1	Sr	Sr1	0.9979	0.99323	0.67706
3	Sr	Sr3	0.50049	0.99763	0.67562
5	Sr	Sr5	0.99794	0.50648	0.67707
7	Sr	Sr7	0.50034	0.5022	0.67557
9	La	La1	0.99745	0.00147	0.44059
11	La	La3	0.49758	0.00029	0.43982
13	La	La5	0.99748	0.49828	0.44058
15	La	La7	0.49753	0.49943	0.43983
17	Co	Co1	0.25184	0.25003	0.61648
19	Co	Co3	0.7538	0.25003	0.61864
21	Co	Co5	0.75008	0.24994	0.38138
23	Co	Co7	0.24617	0.24997	0.38403
25	Co	Co9	0.25153	0.25001	0.50031
27	Co	Co11	0.74877	0.25	0.5002
29	O	O1	0.00219	0.25038	0.60683
31	O	O3	0.74917	0.99973	0.61411
33	O	O5	0.99922	0.25061	0.37703
35	O	O7	0.25273	0.9999	0.61362
37	O	O9	0.49745	0.24958	0.38999
39	O	O11	0.74628	0.49987	0.3862
41	O	O13	0.50037	0.24952	0.62586
43	O	O15	0.25178	0.4997	0.38598
45	O	O17	0.24825	0.00038	0.49988
47	O	O19	0.00083	0.25014	0.51006
49	O	O21	0.24852	0.49962	0.49963
51	O	O23	0.49953	0.24983	0.48965
53	O	O25	0.20957	0.24972	0.67826
55	O	O27	0.78078	0.25012	0.67318
57	O	O29	0.73623	0.24969	0.32633
59	O	O31	0.25633	0.25018	0.32024
61	O	O33	0.2101	0.24969	0.44145
63	O	O35	0.78276	0.25033	0.44253
65	O	O37	0.71229	0.24975	0.55794
67	O	O39	0.29522	0.25037	0.55871
69	S	S1	0.25696	0.24996	0.72672
71	O	O41	0.18038	0.0861	0.74162
73	O	O43	0.18006	0.08686	0.25849
75	O	O45	0.18064	0.41411	0.74153
77	O	O47	0.18003	0.41331	0.25847
79	O	O49	0.45189	0.24989	0.72778
81	O	O51	0.45921	0.2501	0.26277
83	S	S3	0.26876	0.25012	0.27013

LSC with SrO termination, one O_2 adsorbate:

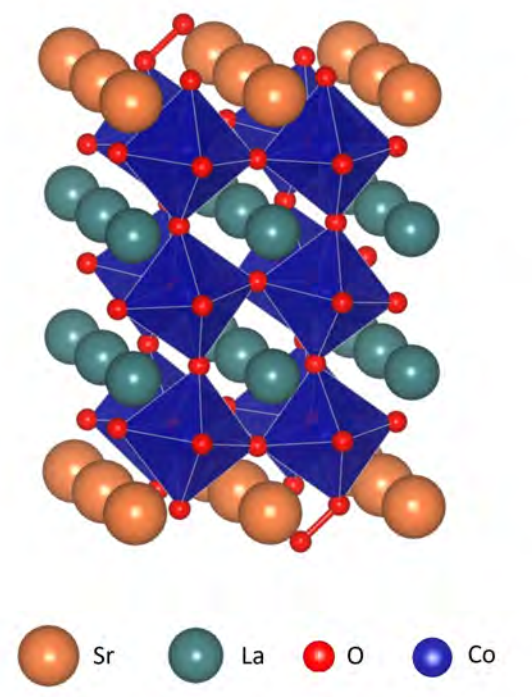

			x	y	z
1	Sr	Sr1	0.98771	0.00901	0.32919
3	Sr	Sr3	0.50211	0.00622	0.32697
5	Sr	Sr5	0.98699	0.49167	0.32914
7	Sr	Sr7	0.50167	0.4956	0.32716
9	La	La1	0.00207	0.99643	0.55934
11	La	La3	0.50199	0.99958	0.56
13	La	La5	0.0019	0.50438	0.55934
15	La	La7	0.50218	0.50147	0.55993
17	Co	Co1	0.74639	0.75004	0.3799
19	Co	Co3	0.24738	0.75011	0.38127
21	Co	Co5	0.25467	0.74997	0.62018
23	Co	Co7	0.7528	0.74998	0.62074
25	Co	Co9	0.74822	0.75005	0.4996
27	Co	Co11	0.25088	0.75002	0.49962
29	O	O1	0.99613	0.7481	0.39079
31	O	O3	0.24293	0.00061	0.38473
33	O	O5	0.00382	0.74793	0.61932
35	O	O7	0.74769	0.00035	0.3852
37	O	O9	0.50368	0.75189	0.61186
39	O	O11	0.25627	0.50029	0.61502
41	O	O13	0.49479	0.75244	0.37351
43	O	O15	0.75241	0.50058	0.61503
45	O	O17	0.75132	0.0001	0.50002
47	O	O19	0.9996	0.74922	0.48966
49	O	O21	0.74936	0.4999	0.50045
51	O	O23	0.49972	0.75099	0.51023
53	O	O25	0.77125	0.75031	0.32278
55	O	O27	0.19888	0.74924	0.32305
57	O	O29	0.2612	0.74996	0.67844
59	O	O31	0.74585	0.74955	0.67807
61	O	O33	0.78303	0.75058	0.55775
63	O	O35	0.21959	0.74945	0.55767
65	O	O37	0.2896	0.75053	0.44071
67	O	O39	0.70639	0.74948	0.44128
69	O	O41	0.33628	0.75107	0.29325

LSC with SrO termination, one O_2 adsorbate and two SO_4^{2-} adsorbates:

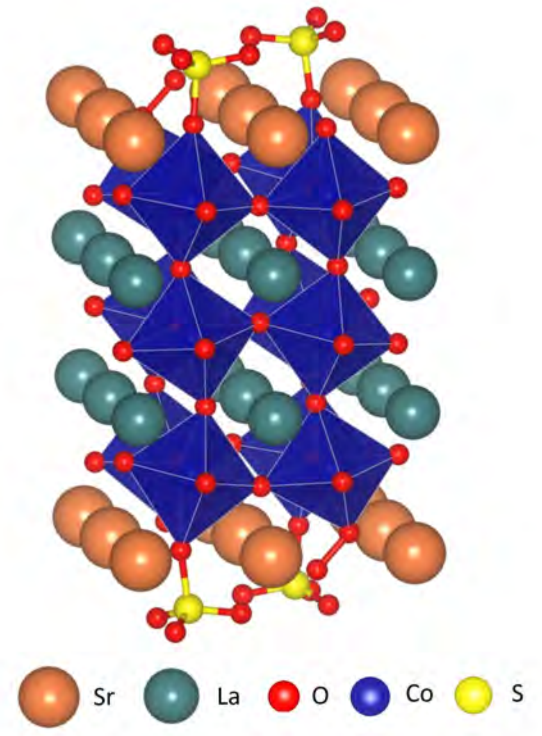

			x	y	z
1	Sr	Sr1	0.98732	0.01965	0.32273
3	Sr	Sr3	0.50828	0.00591	0.32205
5	Sr	Sr5	0.98263	0.47615	0.32163
7	Sr	Sr7	0.5066	0.4967	0.32205
9	La	La1	0.00639	0.99714	0.55871
11	La	La3	0.50363	0.99773	0.56077
13	La	La5	0.0057	0.50423	0.55874
15	La	La7	0.50404	0.50266	0.56078
17	Co	Co1	0.74268	0.74974	0.38401
19	Co	Co3	0.2398	0.7502	0.38432
21	Co	Co5	0.25415	0.75008	0.61814
23	Co	Co7	0.75709	0.75006	0.61578
25	Co	Co9	0.74712	0.74998	0.49971
27	Co	Co11	0.25278	0.74989	0.49971
29	O	O1	0.99264	0.74849	0.3962
31	O	O3	0.24839	0.99997	0.38388
33	O	O5	0.00389	0.74776	0.62235
35	O	O7	0.74685	0.99936	0.38579
37	O	O9	0.50578	0.75227	0.61038
39	O	O11	0.25782	0.49969	0.61467
41	O	O13	0.49421	0.75131	0.37245
43	O	O15	0.74645	0.50078	0.61431
45	O	O17	0.75358	0.99956	0.49987
47	O	O19	0.99928	0.74978	0.49023
49	O	O21	0.75395	0.5005	0.50089
51	O	O23	0.50099	0.75017	0.51118
53	O	O25	0.78484	0.74943	0.32002
55	O	O27	0.16929	0.74377	0.3309
57	O	O29	0.28901	0.75765	0.30077
59	O	O31	0.26427	0.75043	0.67447
61	O	O33	0.748	0.7481	0.68068
63	O	O35	0.79201	0.7525	0.55854
65	O	O37	0.21929	0.74917	0.55733
67	O	O39	0.29466	0.75175	0.44196
69	O	O41	0.69783	0.74914	0.44134
71	S	S1	0.75528	0.75051	0.27043
73	O	O43	0.83765	0.91446	0.25715
75	O	O45	0.82568	0.91215	0.74239
77	O	O47	0.83644	0.58643	0.25678
79	O	O49	0.82547	0.58683	0.74271
81	O	O51	0.56349	0.75104	0.26523
83	O	O53	0.54547	0.74945	0.73833
85	S	S3	0.7363	0.74933	0.73078

LSC with SrO termination and two CO_3^{2-} adsorbates:

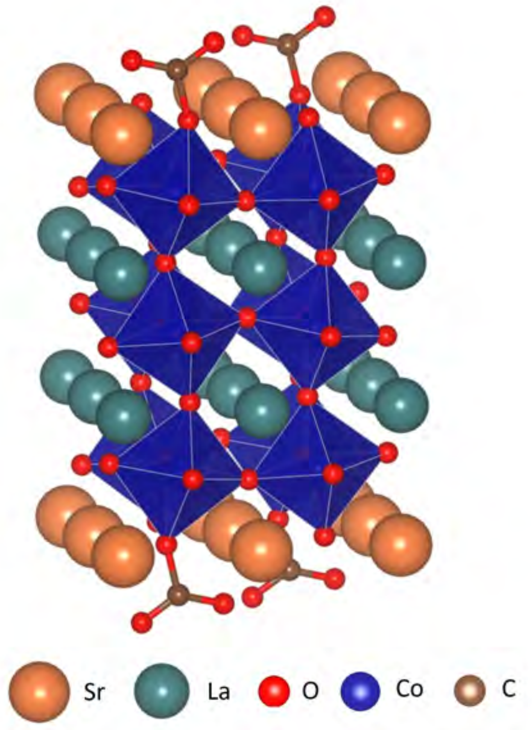

			x	y	z
1	Sr	Sr1	0.99258	0.99443	0.67627
3	Sr	Sr3	0.49274	0.00329	0.67655
5	Sr	Sr5	0.99234	0.50402	0.67663
7	Sr	Sr7	0.49298	0.49518	0.67618
9	La	La1	0.99869	0.00045	0.44022
11	La	La3	0.49876	0.99844	0.44013
13	La	La5	0.99875	0.49834	0.44016
15	La	La7	0.49871	0.50032	0.44018
17	Co	Co1	0.24958	0.25004	0.61711
19	Co	Co3	0.75063	0.25007	0.61937
21	Co	Co5	0.74942	0.24993	0.38062
23	Co	Co7	0.25013	0.24995	0.38294
25	Co	Co9	0.25011	0.25	0.50002
27	Co	Co11	0.74991	0.25	0.50001
29	O	O1	0.99972	0.25204	0.60763
31	O	O3	0.75373	0.00009	0.61472
33	O	O5	0.00118	0.25328	0.37519
35	O	O7	0.24803	0.99981	0.61451
37	O	O9	0.50012	0.24794	0.3922
39	O	O11	0.74635	0.49989	0.38527
41	O	O13	0.49862	0.24674	0.62501
43	O	O15	0.25193	0.50016	0.38547
45	O	O17	0.24967	0.00024	0.50014
47	O	O19	0.00047	0.25054	0.51
49	O	O21	0.25054	0.49976	0.4998
51	O	O23	0.49956	0.24945	0.48998
53	O	O25	0.20033	0.24953	0.67666
55	O	O27	0.76983	0.25032	0.67493
57	O	O29	0.73059	0.24964	0.32504
59	O	O31	0.29742	0.25043	0.32322
61	O	O33	0.20917	0.24948	0.44138
63	O	O35	0.78562	0.25044	0.44206
65	O	O37	0.71403	0.24959	0.55798
67	O	O39	0.29112	0.25055	0.55863
69	C	C1	0.24122	0.24978	0.71931
71	O	O41	0.10797	0.24994	0.74128
73	O	O43	0.40335	0.24983	0.72812
75	C	C3	0.25852	0.2502	0.28041
77	O	O45	0.09684	0.25018	0.27124
79	O	O47	0.39287	0.25002	0.25881

LSC with SrO termination and two CrO_4^{2-} adsorbates:

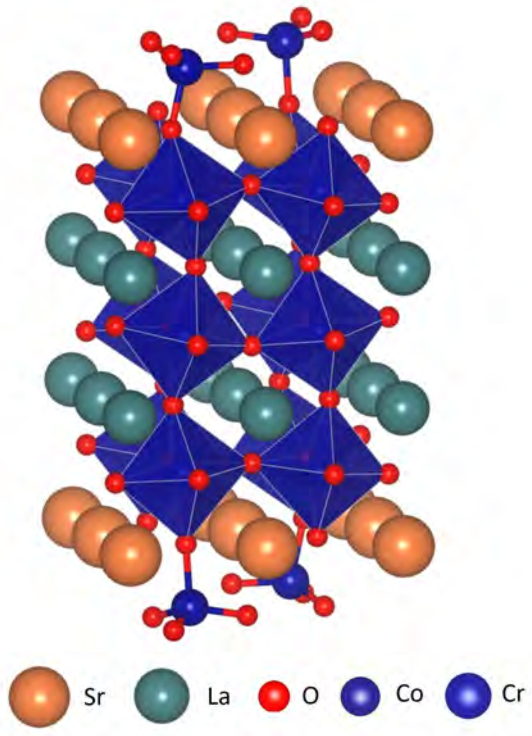

			x	y	z
1	Sr	Sr1	0.99847	0.99336	0.67626
3	Sr	Sr3	0.50031	0.99786	0.67409
5	Sr	Sr5	0.99852	0.50634	0.67628
7	Sr	Sr7	0.50016	0.50193	0.67404
9	La	La1	0.998	0.00201	0.44075
11	La	La3	0.49769	0.00012	0.44
13	La	La5	0.99804	0.49772	0.44074
15	La	La7	0.49764	0.49958	0.44001
17	Co	Co1	0.24871	0.25002	0.61629
19	Co	Co3	0.75331	0.25003	0.61876
21	Co	Co5	0.75015	0.24995	0.38136
23	Co	Co7	0.24645	0.24998	0.38357
25	Co	Co9	0.25135	0.25	0.50023
27	Co	Co11	0.74872	0.25	0.5002
29	O	O1	0.00093	0.25042	0.60639
31	O	O3	0.74915	0.00013	0.61387
33	O	O5	0.99863	0.25067	0.37726
35	O	O7	0.25297	0.99961	0.61343
37	O	O9	0.49785	0.24954	0.3902
39	O	O11	0.74596	0.49958	0.38638
41	O	O13	0.49961	0.24948	0.62582
43	O	O15	0.25189	0.50011	0.38623
45	O	O17	0.24826	0.00027	0.4999
47	O	O19	0.00068	0.25015	0.51008
49	O	O21	0.24855	0.49973	0.49964
51	O	O23	0.49957	0.24982	0.48957
53	O	O25	0.2077	0.2497	0.67487
55	O	O27	0.78113	0.25013	0.67315
57	O	O29	0.73575	0.24968	0.3263
59	O	O31	0.25576	0.25019	0.32378
61	O	O33	0.21022	0.24968	0.44163
63	O	O35	0.78293	0.25035	0.44256
65	O	O37	0.71225	0.24974	0.55795
67	O	O39	0.29535	0.25039	0.55843
69	Cr	Cr1	0.25737	0.24995	0.7273
71	O	O41	0.17242	0.06946	0.74378
73	O	O43	0.17146	0.07009	0.25667
75	O	O45	0.17272	0.43077	0.74368
77	O	O47	0.17142	0.43009	0.25664
79	O	O49	0.47232	0.24986	0.7282
81	O	O51	0.47844	0.25011	0.26147
83	Cr	Cr3	0.26875	0.25013	0.26981

S.I. 2. Work function shift on PCO

In addition to LSC, the effect of sulphate adsorbates on the work function was also investigated for the structurally and chemically different material $Pr_{0.1}Ce_{0.9}O_{2-\delta}$ (PCO). The same fundamental effect was observed for LSC (a) and PCO (b), namely an increase of the work function as a consequence of charge redistribution towards sulphate adsorbates on the surface. However, for PCO, the magnitude of this effect is smaller than for LSC, also the amount of sulphate adsorbates is reduced. We strongly suspect that this is due to the very basic nature of the LSC surface in comparison to the PCO surface.

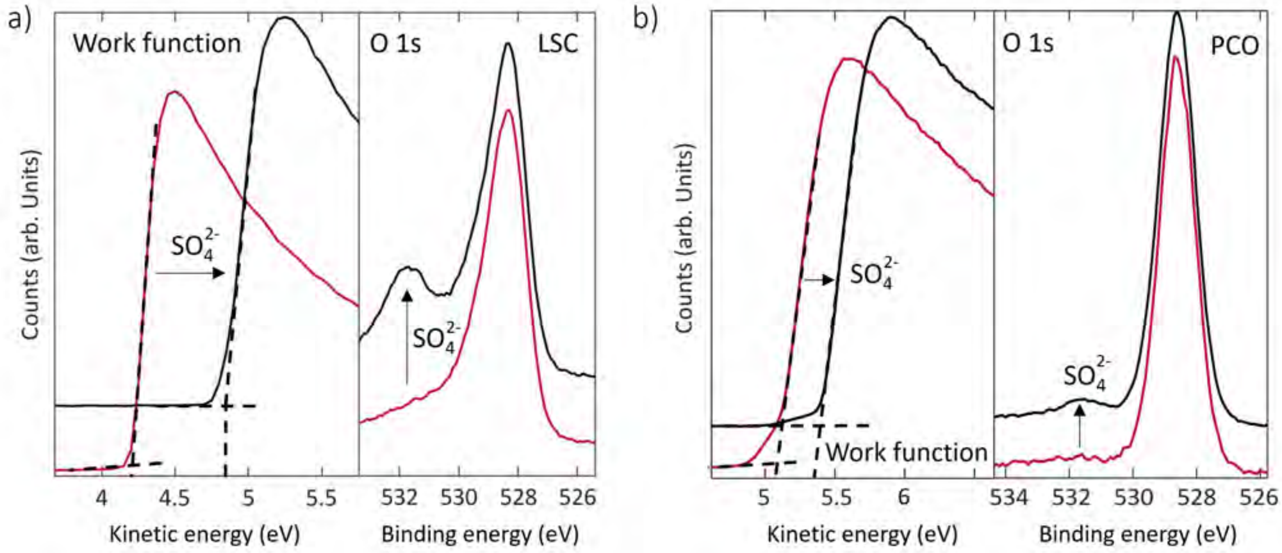

S.I. 3. Structural analysis

The crystalline structure of an LSC thin film was investigated with X-ray diffraction with an Empyrean diffractometer (Panalytical). A 120 nm LSC thin film was deposited on a YSZ single crystal with a GDC20 buffer layer by pulsed laser deposition and the diffractogram is shown in the following figure. The diffractogram clearly shows that LSC grows well oriented in the (001) orientation on a (001) oriented YSZ single crystal with a GDC buffer layer.

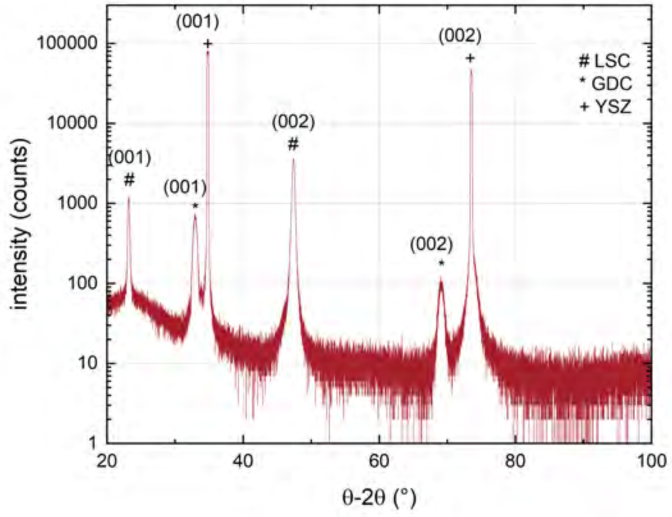

S.I. 4. O_2 dissociation

The dissociation of an O_2 molecule in a surface vacancy on LSC-S0 was investigated computationally. Three positions of the dissociated atom were examined: i) the first Sr bridge position between two surface Sr atoms, ii) the second Sr bridge position in the second lattice direction, iii) the Sr top position on top of a neighbouring Sr atom. The dissociated oxygen atom was constrained in x and y direction and structure relaxation was performed. The following figure shows the relaxed structures:

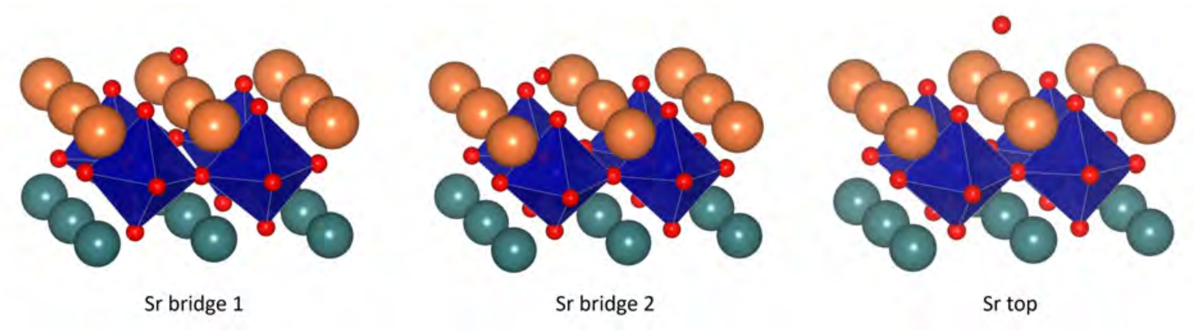

S.I. 5. Reaction energetics

While the convolution of effects on the oxygen exchange kinetics induced by acidic adsorbates severely complicates a quantitative kinetic model for the oxygen exchange reaction rate, one may obtain further insight by approaching the problem from an energetic point of view. As an example, we calculated the start and end stages of the total oxygen exchange reaction

$$O_2(g) + 2V_{O,bulk} \leftrightarrow 2O_{latt} \tag{1}$$

as well as the energy of one reaction intermediate, LSC with an oxygen molecule adsorbed in a surface vacancy (see Fig. **??**). Usually, when the oxygen exchange reaction is discussed experimentally, a rate equation is employed to correlate the reaction rate with the concentrations of participating species [1–4]. For a mechanism in which the rate determining step of the incorporation direction follows the here calculated reaction intermediate, this would read

$$\vec{r} = \vec{k} \cdot [O_{2,surf}] \prod_i \cdot [i] \tag{2}$$

with $\vec{k}$ containing the kinetic barrier of the rate determining step as well as surface potential contributions and $[i]$ denoting additional species which might participate in the reaction step, such as oxygen vacancies or electronic charge carriers. As was already indicated by the previous discussion, the molecular adsorbate on LSC-S0 is energetically much more favourable than on LSC-S2. If one translates this into equilibrium conditions, the concentration of adsorbates will decrease correspondingly and slow down the reaction rate considerably. While this discussion does not take into account the kinetic barriers of the reaction steps (which are drawn schematically in the figure below and which are also altered substantially by SO_4^{2-} adsorbate formation), the energetics of reaction intermediates before and after the rate determining step also affect the overall kinetics of the reaction and may act as a further link between experimental studies in equilibrium conditions and computational investigations of the oxygen exchange reaction. Regarding the here presented calculations, it is also noteworthy that, due to the small supercell and the selective placement of oxygen vacancies, vacancy formation and adsorption energies differ from single vacancy calculations. Hence, this examination is rather a conceptual approach than a quantitative investigation.

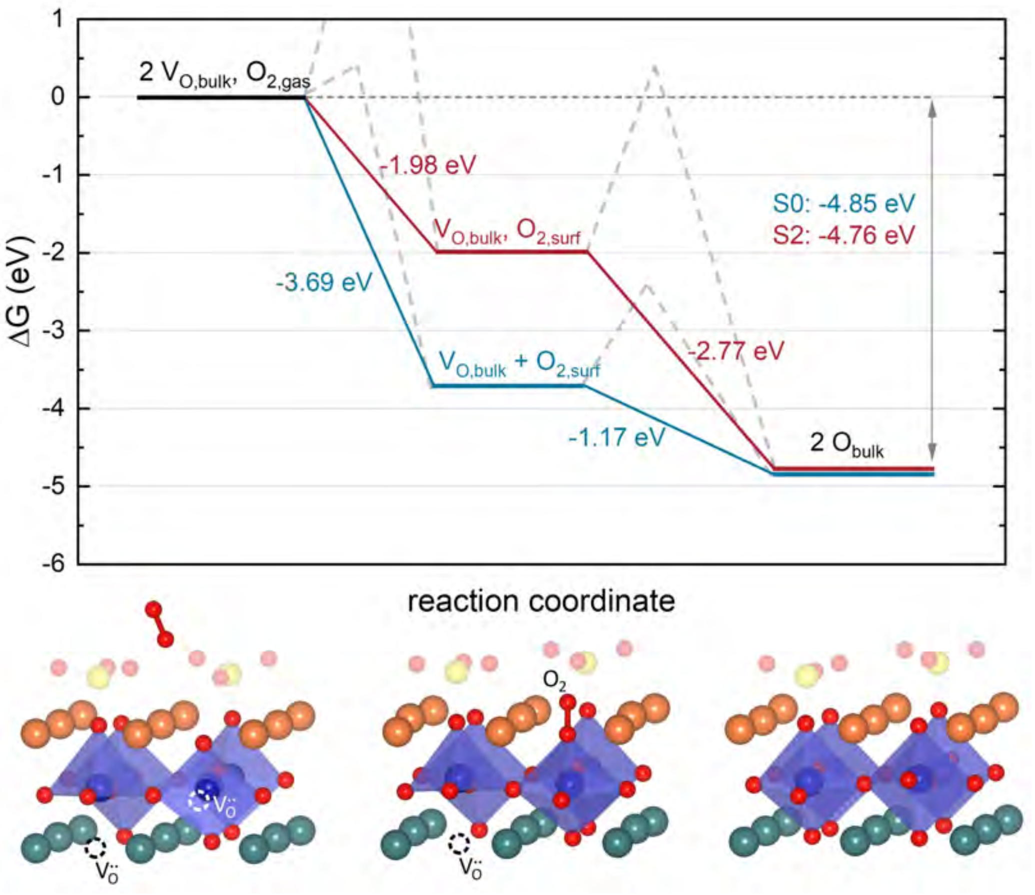

The figure shows the total energy of LSC with two bulk vacancies and one O_2 molecule, of LSC with one bulk vacancy and an oxygen molecule adsorbed in a surface vacancy and of LSC with no oxygen vacancies. The structures below the energy profile correspond to the three reaction steps. Barriers are only drawn schematically, qualitatively estimated from the previously presented results.

References

1. Schmid, A. & Fleig, J. The current-voltage characteristics and partial pressure dependence of defect controlled electrochemical reactions on mixed conducting oxides. *Journal of The Electrochemical Society* **166,** F831 (2019).
2. Adler, S. B., Chen, X. Y. & Wilson, J. R. Mechanisms and rate laws for oxygen exchange on mixed-conducting oxide surfaces. *Journal of Catalysis* **245,** 91–109 (2007).
3. Siebenhofer, M., Riedl, C., Schmid, A., Limbeck, A., Opitz, A. K., Fleig, J. & Kubicek, M. Investigating oxygen reduction pathways on pristine SOFC cathode surfaces by in situ PLD impedance spectroscopy. *Journal of Materials Chemistry A* **10,** 2305–2319 (2022).
4. Mosleh, M., Søgaard, M. & Hendriksen, P. V. Kinetics and mechanisms of oxygen surface exchange on $La_{0.6}Sr_{0.4}FeO_{3-\delta}$ thin films. *Journal of The Electrochemical Society* **156,** B441 (2009).
